# Reviewing the Uncommon Extrahepatic Central Nervous System (CNS) Manifestations of Hepatitis C Virus (HCV) Infection: A Case Report

**DOI:** 10.7759/cureus.76841

**Published:** 2025-01-03

**Authors:** Rabiu Momoh, Benjamin Lawton, Zibran Gaznavee, Amey Kulkarni, Lynden Chiang, John P Dahabreh, Bipin Malkania, Vipal Chawla

**Affiliations:** 1 Critical Care, Medway Maritime Hospital, Gillingham, GBR; 2 Critical Care Medicine, Medway NHS Foundation Trust, Gillingham, GBR; 3 Intensive Care Unit, Medway Maritime Hospital, Gillingham, GBR; 4 Intensive Care Unit, Medway NHS Foundation Trust, Gillingham, GBR

**Keywords:** acute disseminated encephalomyelitis (adem), central nervous system, extrahepatic hepatitis c manifestation, hepatitis c virus, infectious diseases, rare disorders, ring-enhancing brain lesion, status epilepticus, steroid, tumefactive multiple sclerosis

## Abstract

This case report aims to shed more light on the increasingly described scenario of extrahepatic central nervous system (CNS) manifestations of hepatitis C virus (HCV) infection. We present a possible rare case of acute disseminated encephalomyelitis manifesting as ring-enhancing lesions on magnetic resonance imaging (MRI) of the head study conducted on a gentleman in his 50s who presented to the hospital with neurologic features, with an evident demonstration of HCV ribonucleic acid (RNA) in the patient's cerebrospinal fluid sampled. A closer differential of tumefactive multiple sclerosis due to HCV was considered as well. An increasing association of HCV infection with non-Hodgkin's (B-cell) lymphoma, which can potentially affect the brain, is also being noted in the literature. Other common causes of ring-enhancing brain lesions were considered, and attempts were made to exclude a majority of them in this case report.

## Introduction

Hepatitis C virus (HCV) infection, primarily known for its hepatic manifestations, has been implicated in a range of extrahepatic disorders (in up to two-thirds of infected patients with this virus), including autoimmune and neurological conditions [1]. The possible manifestations of the effect of HCV infection on the central nervous system (CNS) are increasingly gaining attention.

The mechanisms by which HCV contributes to neurological disorders are not fully elucidated but are thought to involve immune-mediated processes and direct viral invasion of the neurologic system. Chronic HCV infection induces immune dysregulation, potentially triggering autoimmune conditions such as cryoglobulinemia and systemic vasculitis. These processes may increase CNS vulnerability to inflammation and demyelination, facilitating conditions like acute disseminated encephalomyelitis (ADEM) [2].

We have reviewed two rare possible scenarios in this case report article: ADEM and tumefactive multiple sclerosis due to HCV infection. ADEM represents a diagnostic and therapeutic challenge in neurology due to its complex etiology and overlapping clinical features with other demyelinating diseases. Traditionally associated with common viral infections like measles and mumps, ADEM has more recently been linked to rarer viral triggers, including HCV. Although rare, the occurrence of ADEM in the context of HCV raises critical questions about the underlying mechanisms of immune dysregulation and CNS involvement in chronic viral infections [3].

We present the case of a 57-year-old man, an ex-intravenous drug user, who was diagnosed with HCV infection on index hospital presentation. He was noted to have a high viral load and was admitted to the intensive care unit (ICU) from a medical ward for a status epilepticus. Further evaluation of this patient with magnetic resonance imaging (MRI) of the brain revealed widespread ring-enhancing lesions for which extensive microbiological investigations were done to exclude the common causes of such lesions. There was a rare demonstration of HCV ribonucleic acid (RNA) in the patient's cerebrospinal fluid (CSF). We have also reviewed the existing literature understanding of HCV-associated ADEM and its implications for clinical practice. Non-Hodgkin's (CNS) lymphoma, which is being increasingly associated with HCV infection, was also considered as a differential diagnosis in this case. Other differential diagnoses of these ring-enhancing brain lesions were also reviewed.

## Case presentation

A 57-year-old man was admitted to a regional hospital's emergency after having been found on the floor of his bathroom confused and unable to help himself up. Upon assessment in the emergency room, he was still notably confused and could not recount a history of his possible collapse. 

The patient's past medical history included a history of illicit intravenous drug abuse (he was at the time of admission on daily prescribed methadone), anxiety-depressive disorder, iron-deficiency anemia, past history of intentional self-harm, past treatment for chronic osteomyelitis of the left leg, psoriasis, and abuse of lighter gas (butane gas, inhaled).

On examination, the patient had a black eye on the left side and a small, scabbed cut on his forehead. He expressed pain on palpation of his right hip. His respiratory examination was significant for reduced air entry in both lung bases, with good air entry in the upper lung lobes bilaterally. His cardiovascular examination did not find any abnormalities, nor did his abdominal examination. His neurological examination was significant for his pupils being 3 mm bilaterally and reactive to light. He was able to move all four limbs but not to command.

X-ray studies of his pelvis and right hip and a computed tomography (CT) of his head were all normal studies. His chest X-ray showed right basal consolidation and bilateral minimal pleural effusions. His C-reactive protein (CRP) was noted to be raised at 166.2 ng/l (ref: 0-10 ng/l). He was commenced on intravenous co-amoxiclav. He went on to have two witnessed episodes of generalized tonic-clonic seizures while being observed in the emergency department. The first seizure lasted for one minute and self-terminated, with the second beginning shortly after and lasting 30 seconds. He experienced urinary incontinence during both episodes. He was loaded with intravenous levetiracetam, and further twice-daily doses of this medication were prescribed. He was admitted under the medical team.

The patient was continued on antibiotics and anticonvulsant on the ward. His confusion persisted on the ward. He was awaiting an MRI of the head study. Lumbar puncture was undertaken on day 3 of admission. See the result of serial CSF analyses undertaken on the patient in Table 1.

**Table 1:** Analysis of serial CSF studies undertaken on the patient CSF: cerebrospinal fluid; N/A: not available; HSV: herpes simplex virus; VZV: varicella zoster virus; HCV: hepatitis C virus; RNA: ribonucleic acid

Parameters	Day 3 result	Day 25 result	Day 40 result	Reference range
CSF appearance	Cloudy, orange colored	Clear and colorless	Clear and colorless	Normal appearance: clear, colorless
CSF white cell count	835/ml (polymorphs 60%, lymphocytes 40%)	19/ml (polymorphs 10%, lymphocytes 90%)	4/ml	0-5/ml
CSF red cell count	4080/ml	N/A	215/ml	0/ml
CSF protein	3.8 mg/dl	N/A	3.8 mg/dl	1.5-6 mg/dl
Serum total protein	66 g/l	N/A	N/A	60-80 g/l
CSF glucose	0.9 mmol/l	N/A	N/A	2.77-4.44 mmol/L
Blood glucose	N/A	N/A	N/A	
CSF culture	No growth after incubation for 5 days	No growth after incubation for 5 days	No growth after incubation for 5 days	
CSF microscopy	No organism seen	No organism seen	No organism seen	
CSF virology study	HSV DNA: Not detected	N/A		
	VZV DNA: Not detected			
	Enterovirus RNA: Not detected			
	Overall comment: Probably infective, inflammatory, or a traumatic tap		CSF study from the sample taken on day 40 revealed a CSF HCV RNA count of 292 IU/ml	

Ten days post-admission, the patient had a cluster of seizure episodes and was assessed as being in status epilepticus (multiple recurrent seizure episodes with no full wakefulness in-between, total period lasting about 40 minutes despite an 8 mg total dose of intravenous lorazepam and 1.75 g of intravenous sodium valproate administered). Rapid sequence induction and endotracheal intubation were done on him, after which he was transferred to the ICU. Further treatment with oral lacosamide, levetiracetam, and sodium valproate prevented further acute seizure attacks in the patient.

Further laboratory assessments revealed that the patient was serum anti-HCV antibody reactive, HCV RNA was detected, and his serum HCV RNA viral load was 89,400 IU/ml and had an HCV genotype 3a. He was human immunodeficiency virus (HIV) negative, and his hepatitis B virus screen was negative. He had normal liver function test results (serum albumin: 31 g/l (ref: 35-50 g/l), serum total bilirubin: 14 umol/l (ref: 0-21 umol/l), serum alkaline phosphatase: 77 U/l (ref: 30-130 U/l), serum alanine transaminase: 7 U/l (ref: <50 U/l)), and a CT of the abdomen done on him revealed unremarkable liver, gallbladder, pancreas, spleen, kidneys, and adrenals.

**Figure 1:**
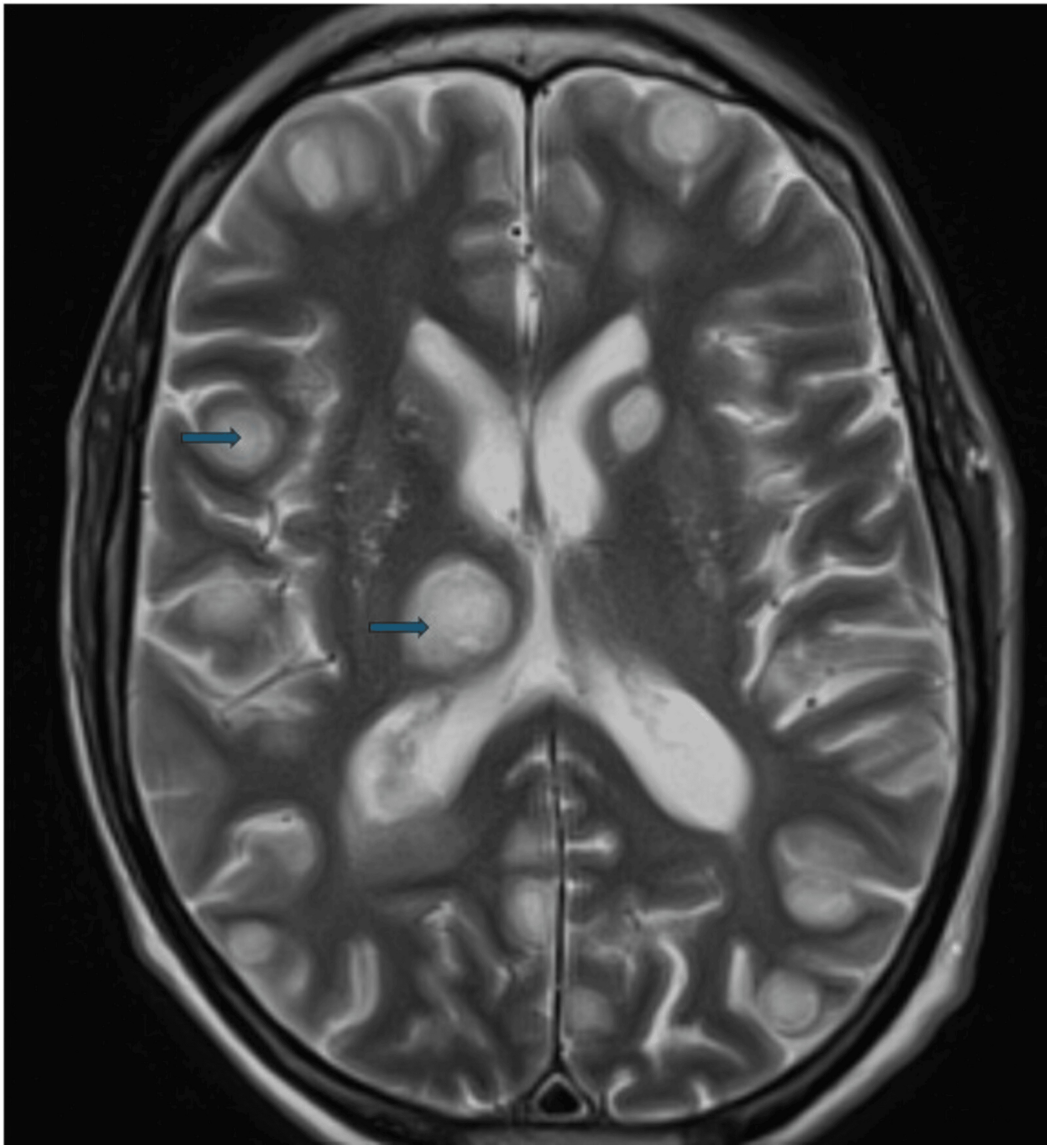
A transverse MRI of the head study section revealing multiple bilateral ring-enhancing lesions MRI: magnetic resonance imaging

An MRI of the head study undertaken shortly after admission to the ICU revealed multiple intracranial lesions demonstrating peripheral ring enhancement and central diffusion restriction (see Figure 1). There were edema involving the left hippocampus and ventriculitis of the left lateral ventricle. A thickened and enhancing pituitary stalk was noted. His toxoplasmosis serology screen was negative, and he had a negative tuberculosis screen.

An MRI of the whole spine study to assess the extent of the brain lesion spread revealed a segmental central T2 hyperintensity lesion with differential diagnosis to include central tumor, transverse myelitis, demyelination in particular ADEM, and a remote possibility of a syrinx. No vertebral metastases or collapse was noted.

A CT of the chest, abdomen, and pelvis study was done on day 11 of hospital admission to exclude malignancy and revealed multiple enhancing polypoid lesions seen over the left colon and sigmoid up to 2.5 cm. Small regional mesenteric lymph nodes up to 12 mm in short axis (suspicious of malignant lesions) were seen. Tumor marker screens, namely, prostate-specific antigen (PSA), carcinoembryonic antigen (CEA), carbohydrate antigen 19-9 (CA 19-9), alpha-fetoprotein (AFP), and beta-human chorionic gonadotrophin (B-HCG), were however negative. Flexible sigmoidoscopy eventually done revealed no acute pathology in the rectum, sigmoid colon, and descending colon. 

**Figure 2:**
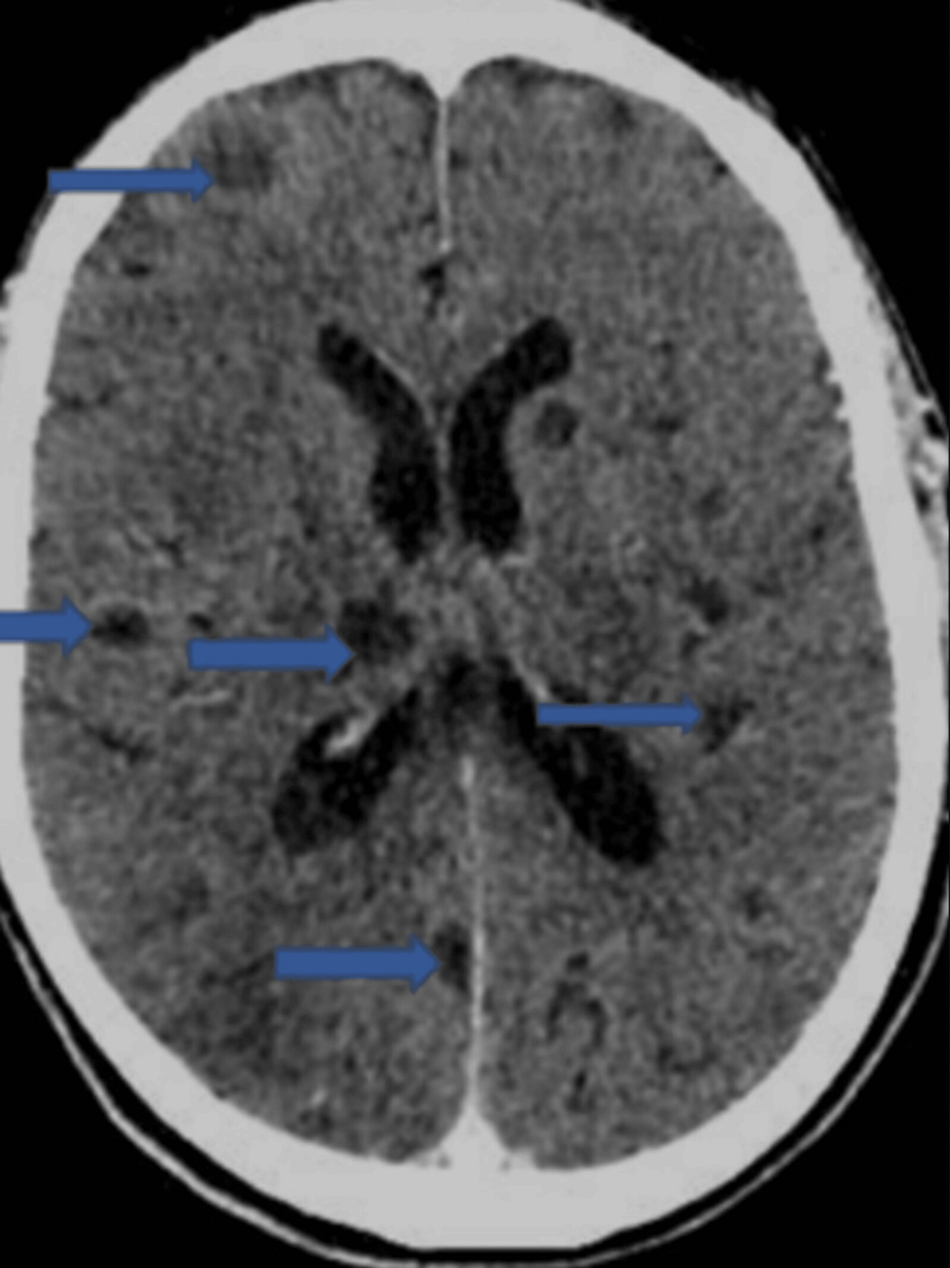
Multiple circular brain lesions The widely disseminated circular lesions are highlighted with the blue arrows

Interval CT of the head with contrast (Figure 2) done on day 13 of hospital admission revealed no interval changes to the round lesions earlier noted on the MRI of the head study earlier conducted.

Serial blood culture studies done in the earlier parts of this patient's hospital admission yielded no growth after up to five days of incubation. Other extensive pathological reports of studies done on the patient are provided in Table 2.

**Table 2:** Pathological test results CSF: cerebrospinal fluid; PCR: polymerase chain reaction; ANA: antinuclear antibodies; ANCA: antibodies against neutrophil cytoplasm

Tests	Result
Serum *Borrelia burgdorferi* IgG and IgM screens	Negative
*Treponema pallidum* serology screen	Negative
CSF *Aspergillus* antigen screen	Negative
CSF beta-D glucan test	Negative
CSF galactomannan test	Negative
Serum *Cryptococcus* antigen screen	Negative
CSF acid-fast bacillus screen	Negative
CSF molecular amplification for tuberculosis	Negative
CSF *Bartonella henselae* and *Bartonella quintana* screens	Negative
CSF *Brucella* screen	Negative
CSF *Toxoplasma* IgG and IgM screens	Negative
CSF 16s rDNA screen	Negative
CSF meningococcal, pneumococcal, and *Haemophilus* PCR screens	Negative
Blood 16s rDNA screen	Negative
ANA and ANCA screens	Negative
Serum light chain screen	Negative
Fecal microbiology assessment	Negative for the presence of *Shigella*, *Salmonella*, and *Campylobacter* and negative for the presence of ova and parasites
Serum electrophoresis	Raised alpha-1 and alpha-2 fractions. No paraprotein was detected
T-SPOT test for tuberculosis	Negative

CSF histopathology evaluation revealed scattered small lymphocytes, occasional macrophages, and some medium-sized degenerate cells like those seen in the CSF direct cytospins. It was thought that the cellular population tends to suggest a chronic inflammatory process rather than a neoplasm.

In light of the patient's history of intravenous drug use, a transthoracic echocardiogram was undertaken to assess for cardio-embolic event as the cause of the patient's multiple brain lesions. This was inconclusive for the presence of vegetations on the mitral and tricuspid leaflets. A transesophageal echocardiogram was then done which excluded the presence of valve vegetations, trivial patent foramen ovale was seen, and no large atrial septal defect was seen (images/videos for these echocardiographic studies are not readily available at the time of this submission).

Intravenous dexamethasone 6.6 mg daily doses were initiated for a week. The patient had several failed sedation holds in the ICU. The electroencephalogram undertaken revealed no unequivocal epileptiform discharges. The findings were in keeping with a mild-moderate non-specific encephalopathy. Tracheostomy was later performed on the patient in view of his prolonged ventilatory requirement and for safe airway control. He was decannulated after 23 days post-tracheostomy insertion. 

CT of the head with contrast done on day 34 of hospital admission revealed numerous scattered enhancing lesions, many of which were new since the previous study and others had increased in size since the previous study with progressive surrounding vasogenic edema. There was progressive compression of the third ventricle with no significant midline shift. An extended treatment course with intravenous dexamethasone 6.6 mg twice daily was re-instituted.

After extensive workup for the differentials of the patient's brain and cervical spinal cord-enhancing lesions, a probable rare diagnosis of ADEM due to HCV infection was thought to be responsible for the patient's pathology. The patient was then considered for the treatment of his HCV infection with Epclusa (a fixed-dose combination of sofosbuvir and velpatasvir).

**Figure 3:**
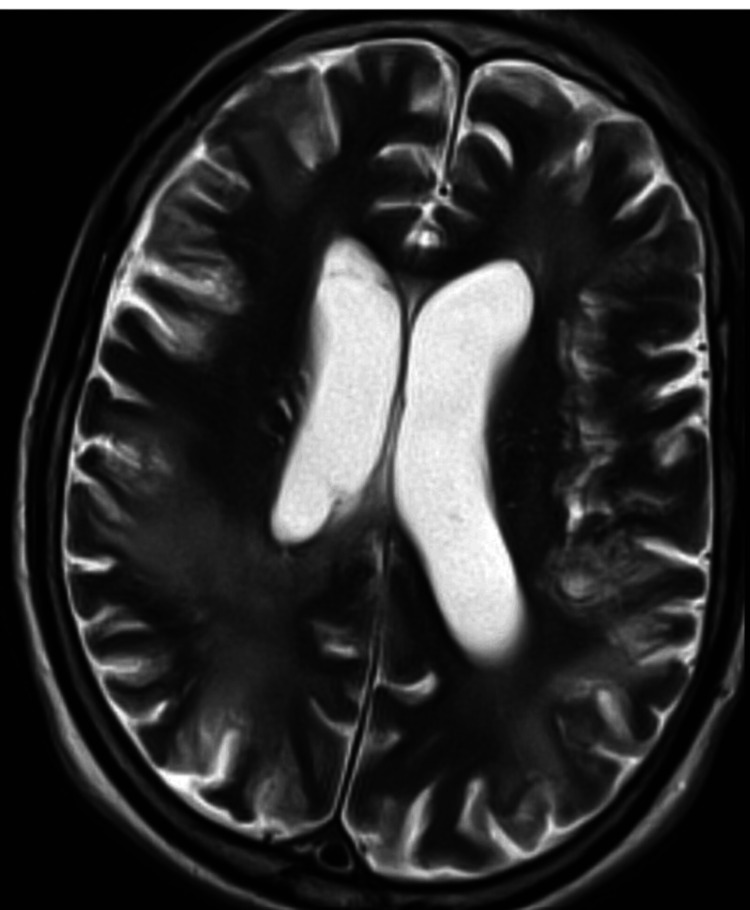
Interval MRI of the brain study showing significant improvement in the burden of the ring-enhancing brain lesions MRI: magnetic resonance imaging

An MRI of the head study done on day 43 of hospital admission noted an improvement as there was an interval decrease in sizes and numbers of known bilateral ring-enhancing lesions on the brain (see Figure 3). MRI of the spine assessment revealed a complete resolution of cervical spinal cord-enhancing lesions. Pathological assessment of a brain biopsy sample of a residual lesion from the right frontal lobe on day 48 was inconclusive, and no further attempts at brain biopsy were undertaken.

Multidisciplinary input from neurology, hepatology, infectious diseases, neurosurgery, and other specialties guided the diagnostic approach and treatment plan in this case. Several antimicrobial combination therapies were considered throughout the course of the patient's stay in the critical care areas (ICU and then stepped down to a high-dependency unit). The patient was eventually stepped down to a neurology-affiliated medical ward under the ongoing care of a neurology team following a successful tracheostomy decannulation on the high-dependency unit. He had a notable cognitive decline because of his illness. He had hypertonia and hyperreflexia in both upper limbs; however, tone and reflex checks in the lower limbs were normal. A follow-up review of the patient on the ward revealed he was on ongoing treatment with sofosbuvir-velpatasvir for HCV and he was continued on intravenous amphotericin, co-trimoxazole, meropenem, and vancomycin. He had completed a treatment course of empirical albendazole. He remained on anti-epileptics and was planned for a weaning steroid regime. 

**Figure 4:**
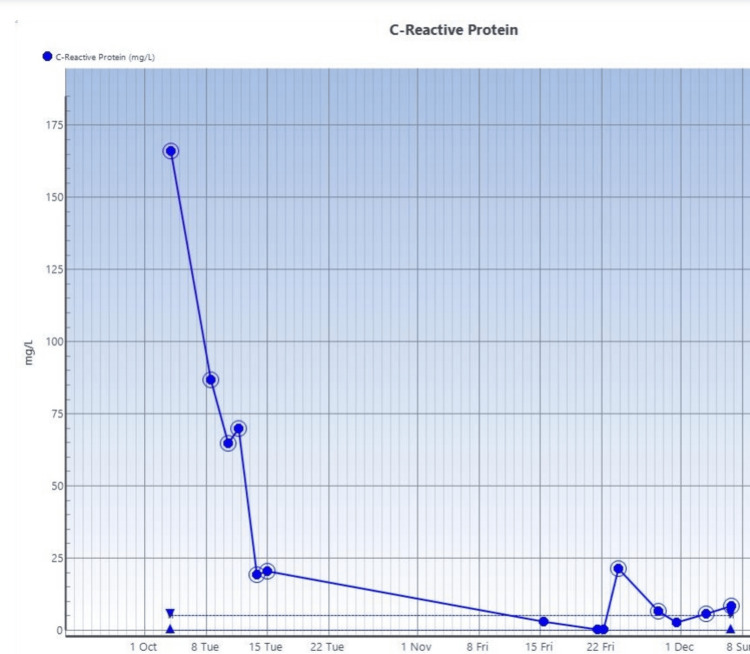
Serial C-reactive protein check Reference range: <10 mg/L

**Figure 5:**
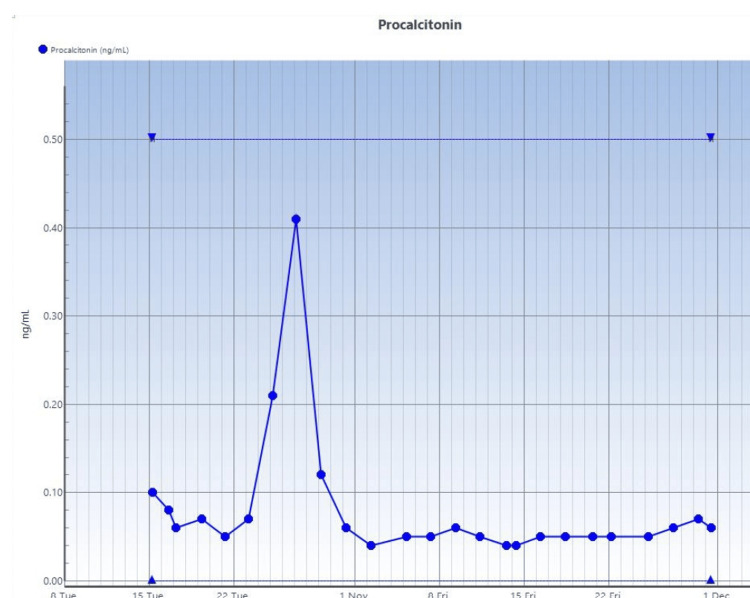
Serial procalcitonin studies Reference range: <0.5 ng/ml

**Figure 6:**
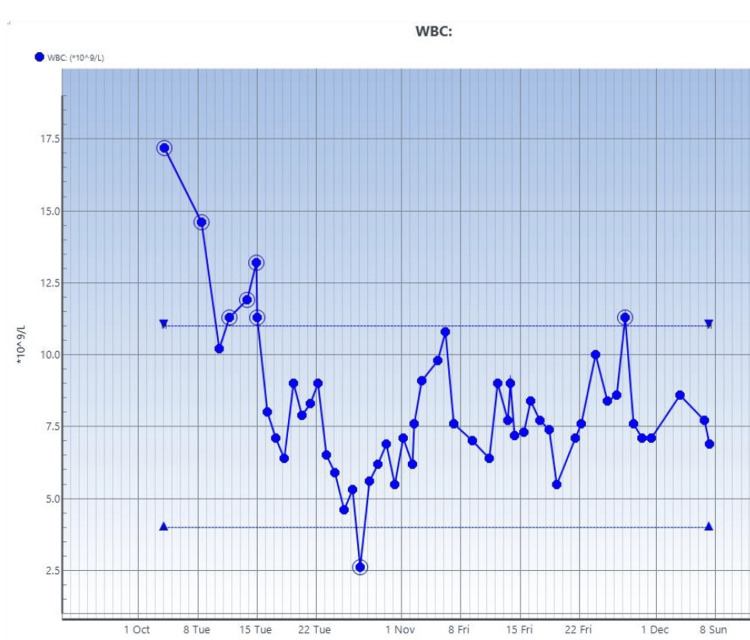
Serial white blood cell count studies Reference range: 4-11×10^9^/l

See serial trends of CRP, procalcitonin, and white blood cell count studies done on the patient in Figure 4, Figure 5, and Figure 6, respectively. 

## Discussion

We have reviewed a possible scenario of ADEM manifesting with widespread ring-like lesions in the brain and cervical spinal cord of a gentleman in his late 50s, an ex-intravenous drug user, with a new diagnosis (and a high viral load) of HCV infection, who presented to the hospital with severe neurological features. Other close differentials of this case include tumefactive multiple sclerosis and possibly non-Hodgkin's CNS lymphoma; both also have topical rare associations with HCV. A possibility of cryptogenic brain abscess of unknown origin was also considered, and the patient had an extended course of antimicrobial cover. An increasing documentation of the extrahepatic manifestations of HCV (including CNS affectation) is noted in the literature. A review of this rare occurrence that could mimic other commonly known CNS pathologies or infections, thereby posing a diagnostic dilemma, is presented below.

ADEM affects about eight per million people per year. Children and adolescents are more affected by ADEM, though it has been reported in all age groups and shows no sex preponderance. It has been associated with a higher incidence in winter and spring months, possibly coinciding with higher viral infections during these periods. The mortality rate for ADEM may reach up to 5%; however, complete recovery is observed in 50-75% of instances, with survival rates improving to 70-90% when accounting for minor residual disabilities. The typical recovery period from ADEM flare-ups ranges from one to six months [3]. About half of patients with chronic HCV infection develop neurologic and neuropsychiatric complications [4]. However, literature evidence of case reports or case series describing ADEM complicating HCV infection are few (less than 10).

The pathogenesis of ADEM in the context of HCV infection remains multifaceted, involving two primary mechanisms: autoimmune-mediated CNS damage and direct viral invasion. Chronic HCV infection may dysregulate the immune system, leading to an autoimmune response that targets CNS myelin. This could occur via molecular mimicry, where viral antigens resemble myelin proteins such as myelin basic protein (MBP), causing an aberrant immune attack on the CNS. Immune dysregulation due to chronic inflammation or cryoglobulinemia may further exacerbate this autoimmune response, contributing to the characteristic demyelination seen in ADEM [5]. Although less common, direct invasion of the CNS by HCV has been documented. Studies detecting HCV RNA in brain tissue support this mechanism, suggesting that the virus may cross the blood-brain barrier. Viral invasion can incite localized inflammation, leading to demyelination and the development of ADEM-like pathology [6].

ADEM typically presents with rapid-onset neurological symptoms, including confusion and altered mental status, motor deficits such as weakness or paralysis, ataxia and other coordination difficulties, and sensory impairments or disturbance. In HCV-associated ADEM, these symptoms may emerge more gradually, making diagnosis challenging. Neuroimaging, particularly MRI, is essential for identifying the hallmark features of ADEM: widespread lesions in the white matter, often with gadolinium enhancement indicative of active inflammation. However, these findings can overlap with other demyelinating diseases, such as multiple sclerosis, complicating differential diagnosis [7].

Diagnosing ADEM in the context of HCV infection requires a thorough clinical evaluation and targeted laboratory investigations. Serologic testing for anti-HCV antibodies and HCV RNA via polymerase chain reaction (PCR) is crucial to confirm the presence of HCV infection [8]. CSF analysis may reveal typical findings in ADEM including elevated protein levels and lymphocytic pleocytosis. The detection of HCV-specific antibodies or RNA in the CSF strongly suggests CNS involvement by HCV [9]. There were however a significant polymorph pleocytosis, an elevated red cell count, a normal protein level, a low CSF glucose level, and a sterile culture in the first CSF sample analyzed in the index case under review. The second CSF sample undertaken on day 25 of the patient's hospital stay revealed a moderately elevated CSF white cell count but revealed a lymphocytic preponderance. Multiple CSF studies were undertaken as further CSF samples were required to exclude other differential diagnoses in this case. MRI findings consistent with demyelination, combined with clinical and laboratory data, are critical for establishing a diagnosis of ADEM.

ADEM has been recognized as one of the possible clinical causes of anti-myelin oligodendrocyte glycoprotein (anti-MOG)-associated encephalomyelitis. It is however unknown if all ADEM cases would be positive for anti-MOG autoantibody [3]. The demonstration of this antibody in the index patient's CSF would have added a more confirmatory assessment of ADEM in our patient. His clinical assessment, MRI demonstration of widespread white matter brain lesions, and evident demonstration of HCV RNA in his CSF added weight to a possible assessment of ADEM due to HCV infection.

Serum electrophoresis study was undertaken in the patient under review, and he had raised alpha-1 and alpha-2 fractions. This suggests a possible ongoing inflammatory or infective state or the presence of malignancy. However, no paraprotein was detected, and assessments for the presence of malignancy in this patient were negative. CSF electrophoresis would have been a further useful assessment to have been done in this case. The demonstration of oligoclonal band negativity on CSF electrophoresis or the finding of a mirrored pattern of oligoclonal bands in CSF and serum electrophoresis is more typical of ADEM. CSF MBP concentration level is noted to be frequently elevated in ADEM, but this has been described as a non-specific finding [10].

Given the non-specific nature of symptoms and overlap with other CNS disorders, a multidisciplinary approach involving neurologists, infectious disease specialists, and radiologists is often required. The management of HCV-associated ADEM involves addressing both the autoimmune CNS inflammation and the underlying viral infection. High-dose corticosteroids are the first-line treatment to reduce CNS inflammation and facilitate the recovery of demyelinated areas. For cases unresponsive to steroids, second-line therapies such as intravenous immunoglobulin (IVIg) or plasmapheresis may be used [11]. The advent of direct-acting antivirals (DAAs) has revolutionized HCV treatment, achieving high rates of sustained virological response (SVR). These agents not only control viral replication but may also reduce immune dysregulation and prevent further neurological complications [12]. In cases complicated by cryoglobulinemia or systemic vasculitis, immunosuppressive agents like rituximab or cyclophosphamide may be necessary to control systemic inflammation and minimize CNS damage [13]. Multidisciplinary management was used in the care of the patient under review. The patient had an extended treatment course with steroid. A fairly delayed initiation of treatment for HCV infection was on the consideration that other possible differential diagnoses were likely the cause of the multiple ring-like brain lesions the patient had and needed exclusion. It was thought that the initiation of such treatment while the patient was deemed critically ill in the ICU could be counterproductive. Treatment with sofosbuvir-velpatasvir was eventually settled on for the treatment of his HCV infection.

The prognosis for ADEM associated with HCV varies. Many patients experience significant neurological recovery, particularly when treatment is initiated early. However, long-term sequelae, such as residual motor weakness or cognitive deficits, may persist in some cases. The presence of comorbid conditions, such as cryoglobulinemia or systemic vasculitis, can further complicate recovery. Early recognition and timely intervention are critical for optimizing outcomes. The availability of effective antiviral therapies, coupled with immunomodulatory treatments, has improved the prognosis for patients with this rare but serious condition.

Another rare terminological description of the CNS manifestation of HCV infection is tumefactive multiple sclerosis. Mader et al. described the finding of brain biopsy-confirmed tumefactive white matter lesions in a 17-year-old female patient with multiple sexual partners and a history of illicit drug injections who was found on their assessment to be HCV infection positive. The histological finding from the stereotactic brain biopsy conducted on their patient revealed the presence of microgliosis, reactive gliosis, glial atypia, and scattered gemistocytes. They acknowledged the rare association of HCV with tumefactive multiple sclerosis and suggested that the absence of encephalopathy in their patient negated her diagnosis as ADEM (a similar which is usually accompanied by encephalopathy) [14]. The HCV genotype in their patient is 2a/2c, while in our index case report the patient's HCV genotype is 3a. The viral load in the case presented by Mader et al. was 15,217 IU/ml, while the viral load detected in our index case report was 89,400 IU/ml. Our patient appeared to have more multifocal brain lesions compared to theirs. The presence of encephalopathy in our index case tips the potential diagnosis in our case towards ADEM. Mader et al. went on to suggest that CNS myelin damage via autoimmune activities could be the result of HCV genotype 2a/2c. They treated their patient with high-dose steroid and interferon beta-1a.

McGee and Minagar, in a case report, also shared a similar perspective on the role of HCV in causing multifocal tumefactive white matter brain lesions. They suggested that these brain lesions (which could be oval-shaped, well-circumscribed, and ring-enhancing) could resemble abscesses or tumors [15]. In our index case, efforts were made to exclude the brain lesions as secondary brain metastasis via tumor marker studies and the conduct of CT of the chest, abdomen, and pelvis studies with contrast. Multiple CSF samples were analyzed in the microbiology lab, and the only positive microbiological demonstration was the presence of anti-HCV antibodies in the CSF. Our patient was HIV negative, and extensive CS studies for common culpable organisms that cause ring-enhancing lesions like toxoplasmosis, cryptococcosis, fungal infections, syphilis, and tuberculosis among others were negative.

Ring-enhancing lesions in the brain are striking findings in imaging studies and can result in a major diagnostic challenge. Their causes are diverse, spanning from infections, malignancies, autoimmune disorders, and vascular conditions. To reach an accurate diagnosis, clinicians must combine imaging studies with a patient's clinical history and other diagnostic tools. Clinical context, such as geographic exposure, the individual's immune status, and systemic symptoms, is critical for narrowing down possible diagnoses. A list of the long differentials for this case below was considered, and attempts were made to exclude as much of these by multidisciplinary reviews and by extensive investigations done on the patient's blood, CSF, urine, and attempted brain tissue biopsy.

## Conclusions

This case underscores the challenges of diagnosing and managing multifaceted neurological and systemic infections, emphasizing the need for multidisciplinary collaboration and adaptability in treatment strategies when faced with rare presentations of encephalomyelitis or multiple disseminated brain ring-enhancing lesions. Our case report adds further evidence to consider HCV infection (alongside other commoner causes) as a possible etiological factor in the management of patients with multifocal or ring-enhancing lesions on the brain. We demonstrated a high serum viral load in the case under review and a demonstration of HCV RNA in the patient's CSF sample. Though uncommon, our index case and other cases noted in the literature suggest that ADEM or tumefactive multiple sclerosis due to HCV infection can be associated with significant morbidity/mortality. Multidisciplinary management and the prompt initiation of treatment can help to prevent untoward outcomes. Further research into this uncommon extrahepatic HCV manifestations is suggested.

## References

[1] Cacoub P, Comarmond C, Domont F, Savey L, Desbois AC, Saadoun D (2016). Extrahepatic manifestations of chronic hepatitis C virus infection. Ther Adv Infect Dis.

[2] Origgi L, Vanoli M, Carbone A, Grasso M, Scorza R (1998). Central nervous system involvement in patients with HCV-related cryoglobulinemia. Am J Med Sci.

[3] Wikipedia contributors. (2024, December 4 (2024). Acute disseminated encephalomyelitis. https://en.wikipedia.org/w/index.php?title=Acute_disseminated_encephalomyelitis&oldid=1261111343.

[4] Adinolfi LE, Nevola R, Lus G (2015). Chronic hepatitis C virus infection and neurological and psychiatric disorders: an overview. World J Gastroenterol.

[5] Sacconi S, Salviati L, Merelli E (2001). Acute disseminated encephalomyelitis associated with hepatitis C virus infection. Arch Neurol.

[6] Bolay H, Söylemezoğlu F, Nurlu G, Tuncer S, Varli K (1996). PCR detected hepatitis C virus genome in the brain of a case with progressive encephalomyelitis with rigidity. Clin Neurol Neurosurg.

[7] Menge T, Hemmer B, Nessler S (2005). Acute disseminated encephalomyelitis: an update. Arch Neurol.

[8] Dawson TM, Starkebaum G (1999). Isolated central nervous system vasculitis associated with hepatitis C infection. J Rheumatol.

[9] Sim JE, Lee JB, Cho YN, Suh SH, Kim JK, Lee KY (2012). A case of acute disseminated encephalomyelitis associated with hepatitis C virus infection. Yonsei Med J.

[10] Brenton JN (2024). Acute disseminated encephalomyelitis workup. Medscape.

[11] Anilkumar AC, Foris LA, Tadi P (2024). Acute disseminated encephalomyelitis. StatPearls [Internet].

[12] Baumert TF, Berg T, Lim JK, Nelson DR (2019). Status of direct-acting antiviral therapy for hepatitis C virus infection and remaining challenges. Gastroenterology.

[13] Montero N, Favà A, Rodriguez E, Barrios C, Cruzado JM, Pascual J, Soler MJ (2018). Treatment for hepatitis C virus-associated mixed cryoglobulinaemia. Cochrane Database Syst Rev.

[14] Mader EC Jr, Richeh W, Ochoa JM, Sullivan LL, Gutierrez AN, Lovera JF (2015). Tumefactive multiple sclerosis and hepatitis C virus 2a/2C infection: dual benefit of long-term interferon beta-1a therapy?. J Neurol Sci.

[15] McGee J, Minagar A (2015). Co-existence of tumefactive MS and hepatitis C: a need for further screening and new therapeutic challenge. J Neurol Sci.

